# Amino acids and their metabolites as potential biochemical markers in *postmortem* vitreous humour

**DOI:** 10.1007/s00414-025-03552-9

**Published:** 2025-07-02

**Authors:** Laura Franke, Hannah Ihle, Kristina Rieger, Viviane Stammer, Senta Niederegger, Dirk K. Wissenbach, Frank T. Peters, Gita Mall

**Affiliations:** 1 Jena University Hospital, Institute for Forensic Medicine, Friedrich Schiller University, Jena, Germany; 2 Perioperative Medizin und Notfallmedizin Helios Klinikum Erfurt, Klinik für Anästhesiologie, Erfurt, Germany; 3 Institute for Forensic Medicine, Heidelberg, Germany

**Keywords:** Putrefaction, *Postmortem* decay, Bacterial metabolism, Vitreous humour, LC-HRMS, Amino acids, Biogenic amines, Acetylated amino acid metabolites

## Abstract

**Background:**

Putrefaction is the microbial metabolism/degradation of autolysis end products like amino acids. The aim of this study was to identify and investigate amino acids and their bacterial degradation products/metabolites by LC-HRMS as possible markers of putrefaction.

**Methods:**

Pig eyes (*n* = 60) were stored in conical centrifuge tubes and petri dishes within a climate chamber at 25°C, 65% humidity over four days. Each day, eyes were macroscopically examined and vitreous humour was drawn from up to six eyes. Twenty-one amino acids/metabolites (lysine, histidine, arginine, *N*^*2*^-acetyllysine, *N*^*2*^-acetylarginine, *N*^*6*^-acetyllysine, *N*-acetylcadaverine, tyrosine, *N*-acetylagmatine, tyramine, phenylalanine, *N*^*2,6*^-diacetyllysine, 2-phenylethylamine, tryptophan, kynurenic acid, *N-*acetyltyrosine, *N*-acetyltyramine, *N*-acetylphenylalanine, *N*-acetyltryptophan, *N*-acetyl-2-phenylethylamine, *N*-acetyltryptamine**)** were monitored by LC-HRMS.

**Results:**

Opacification was observed and eyes stored on petri dishes dried out faster and turned dark compared to eyes stored in centrifuge tubes. Amino acids were universally present and showed a significant increase by up to 46-fold from day 0 to 4, except for arginine (no trend observed). Amino acid metabolites were first detected on day 2 with exception of *N*^*2*^-acetyllysine, *N*^*2*^-acetylarginine, and *N*^*6*^-acetyllysine (detected on day 0). All analytes showed a marked increase by days 3 and 4, especially kynurenic acid and *N*-acetylated compounds. Among the six eyes of each storage condition/day high variability was observed, between centrifuge tube and petri dish storage few variability was found.

**Conclusion:**

LC-HRMS analysis of amino acids and their metabolites in vitreous humour could be a promising tool to evaluate the putrefaction status. However, further studies are required to better understand inter-individual metabolic differences and influencing environmental factors.

**Supplementary Information:**

The online version contains supplementary material available at 10.1007/s00414-025-03552-9.

## Introduction

Certain *postmortem* (PM) decay processes may allow determination of the time of death and *postmortem* interval (PMI), which is one of the key tasks in legal medicine [1, 2]. However, an exact determination can be difficult due to non-linear progression of the underlying processes and multiple exogenous (e.g. temperature, humidity) and endogenous (e.g. age, disease) influencing factors [3, 4].

Several methods for the estimation of short PMIs have been established. Physical parameters, especially body cooling, and signs of death (e.g. rigor mortis) [5–10] as well as supravital reactions [11, 12] are routinely used [1]. Even though these signs result from biochemical changes in the body after death [13, 14], chemical death time estimation methods play a marginal role in daily forensic practice [1, 3, 15].

**Fig. 1:**
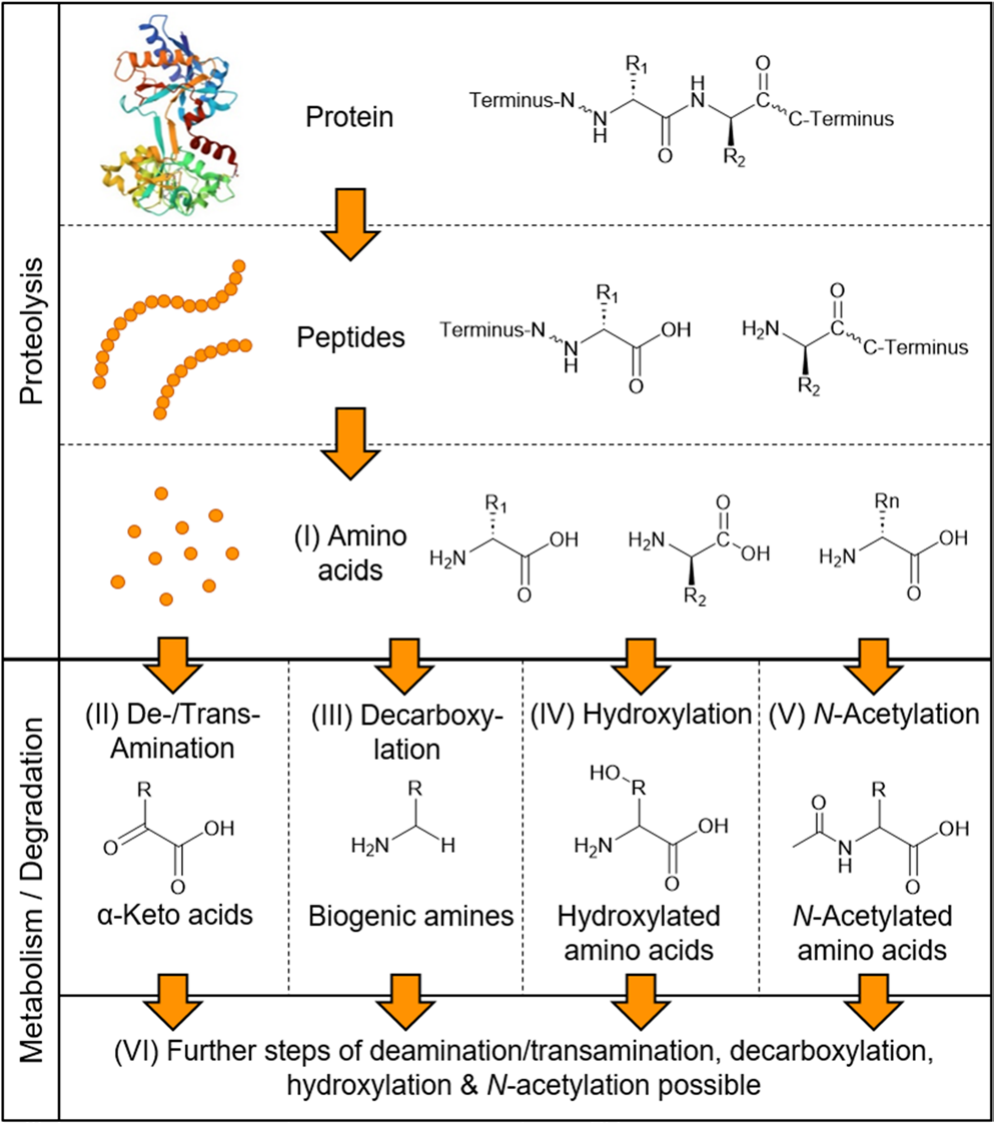
Schematic overview of protein degradation and further metabolism by microbes during autolysis and putrefaction. Protein structure was obtained from RCSB Protein Data Base: 10.2210/pdb1BP5/pdb according to Jeffrey et al*.*

For PM decay (decomposition) different processes were described in the literature such as *autolysis* and *putrefaction* [4]. PM autolysis is defined as cell/macromolecule degradation by intracellular enzymes [4, 16, 17]. The end products of the autolysis process may serve as substrates and nutrients for (micro)organisms, which drive putrefaction [4, 18]. Putrefaction is influenced by the metabolism of anaerobic bacteria inside the body but also aerobic bacteria on the body surface [17, 19]. Proteolysis may be facilitated by autolysis with respect to body-own proteases, such as calpains [20], or microbial proteases. Figure 1 shows the protein degradation to (I) amino acids (AA) and further microbial metabolism.

Those AA can be further metabolised/degraded/catabolised by (II) transamination/deamination, (III) decarboxylation, (IV) hydroxylation or (V) *N*-acetylation (Fig. 1) [21]. At the end of the degradation cascade, volatile biogenic amines, ammonia, and carbon dioxide are formed [19]. Some of these compounds such as cadaverine (Cad) or putrescine can cause the typical decomposition odour [19, 22–24]. Due to improvements in analytical methodology, biochemical biomarkers from different compound classes have come into focus for estimation of the PMI [3, 13, 25]. Promising biochemical biomarkers have been found already for kidney tissue and blood [13, 25, 26]. In a previous study, the *N*-acetylated biogenic amine *N*-acetyltyramine (NATrm) was identified in PM heart blood [27]. Besides, PM changes of the eyes and vitreous/aqueous humour are under research [14, 28–30]. In vitreous and/or aqueous humour the increase in potassium concentrations with later PMI was intensively studied [30–34], but also small endogenous biomolecules from different compound classes like sugars with glucose, RNA/nucleotide metabolism like uracil and hypoxanthine; AA such as aspartate, glutamate, glutamine, glycine, lysine (Lys), phenylalanine (Phe), tryptophan (Trp), tyrosine (Tyr); amino acid metabolites (AAM) including taurine and γ-aminobutyric acid and many more compounds were investigated in several studies including metabolomics approaches [14, 29, 30, 35–38].

Intraocular fluids are believed to be less prone to bacterial contamination (putrefaction), environmental factors and even autolysis due to the anatomic isolation of the eyes with their blood-retinal barrier limiting substance exchange between the eyes and other body fluids/compartments [1, 30, 39–41]. Thus, PM biochemical changes proceed more gradually and the eyes/intraocular fluids have some advantages over other biomatrices when investigating the (early) PM period from hours to several days [14, 31–33, 39, 41–43].

The aim of the presented study was to characterise the putrefaction process by monitoring the decay or formation of small (< 1,000 Da) endogenous biomolecules. A particular focus was on AA degradation cascades and corresponding AA degradation/metabolism products (Fig. 1). Pig eyes were used as model matrix and ocular changes in appearance were correlated to the observed AA/AAM patterns.

## Materials and methods

### Chemicals

Water, acetonitrile (ACN), methanol and formic acid were obtained from Fisher Chemical (Schwerte, Germany) at the highest available level of purity (LC-HRMS-grade). Ammonium formate was provided by Sigma-Aldrich (Steinheim, Germany). Analytical standards were purchased for Lys (Sigma-Aldrich Chemie GmbH, Steinheim, Germany), histidine (His) (European Pharmacopoeia (EP) Reference Standard H0750000, Council of Europe– EDQM, Strasbourg, France), Cad (Sigma-Aldrich Chemie GmbH, Steinheim, Germany), arginine (Arg) (Sigma-Aldrich Chemie GmbH, Steinheim, Germany), agmatine (Agm) (Sigma-Aldrich Chemie GmbH, Steinheim, Germany), histamine (Htm) (Sigma-Aldrich Chemie GmbH, Steinheim, Germany), octopamine (Oct) (Sigma-Aldrich Chemie GmbH, Steinheim, Germany), *N*^6^-acetyllysine (N6ALys) (Sigma-Aldrich Chemie GmbH, Steinheim, Germany), *N*-acetylhistamine (NAHtm) (Sigma-Aldrich Chemie GmbH, Steinheim, Germany), Tyr (Sigma-Aldrich Chemie GmbH, Steinheim, Germany), tyramine (Trm) (Sigma-Aldrich Chemie GmbH, Steinheim, Germany), Phe (Sigma-Aldrich Chemie GmbH, Steinheim, Germany), 2-phenylethylamine (PEA) (Sigma-Aldrich Chemie GmbH, Steinheim, Germany), Trp (Merck, Darmstadt, Germany), tryptamine (Try) (Sigma-Aldrich Chemie GmbH, Steinheim, Germany), NATrm (TRC/LGC Standards GmbH, Wesel, Germany), *N*-acetyl-2-phenylethylamine (NAPEA) (Chemos GmbH & Co_KG, Altdorf, Germany), and *N*-acetyltryptamine (NATry) (Iris Biotech GmbH, Marktredwitz, Germany).

### Biomaterial and storage conditions

Pig eyes (usually discarded as waste products) were obtained fresh on slaughter day from a local butcher (*n* = 60). Pigs were approximately 180 days old with a weight of about 110 kg, were equally distributed male and female, came from different breeders and were slaughtered all by the same procedure (electrical anaesthetization and exsanguination) for the meat industry. Adherent soft tissue was detached from the eyes and they were placed in a climate chamber (settings: 25°C; 65% humidity; without ventilation) on petri dishes (*n* = 30) and truncated 50 mL self-standing conical centrifuge tubes (*n* = 30) for up to four days. A close-by data logger monitored the temperature and humidity. Each day, beginning with the start of the experiment on day 0 (d0) when the eyes were fresh to d4 when the eyes showed macroscopic signs of putrefaction, vitreous humour was drawn from six eyes, respectively, if available (eyes were only punctured once). On d0 the PMI was 10 h at the first sample collection and collection intervals varied between 21 h and 27 h in the following days. Sampling of vitreous humour occurred through punctuation of the vitreous body via hollow needle. Samples were stored at −80°C.

### Sample Preparation

All samples were processed by protein precipitation within one batch. In detail, 10 µL of vitreous humour were precipitated with 50 µL of precipitation solution (ACN: MeOH; 4:1; *v:v*). The mixture was vortexed (30 s) and centrifuged (2 min) at 9447 x g. Ten µL of the supernatant were diluted by addition of 40 µL mobile phase A (MP A; 10 mM ammonium format, 0.1% formic acid) and analysed by liquid chromatography coupled to high-resolution mass spectrometry (LC-HRMS). Additionally, a pooled vitreous humour sample (stored frozen at −80°C until analysis; from six independent pig eyes) was prepared by the same protocol and used as control to check for signal drift while LC-HRMS measurement.

### Acetylation procedure to produce analytical standards

Analytical standards of Lys, His, Cad, Arg, Agm, Tyr, Phe, and Trp were used to produce their acetylated metabolites. Ten µL of a 1 µg/mL solution of each analytical standard were evaporated under a gentle stream of nitrogen at 60°C. A half-molar acetylation mixture (acetic anhydride:pyridine 3:2, *v:v*) was added to the dried residues of each analytical standard, respectively. After 1 min heating in the microwave at 700 W, the acetylation mixture was completely evaporated under a gentle stream of nitrogen at 60°C. The residue was dissolved in 50 µL of a 3:1 (*v:v*) mixture of MP A and B (ACN, 0.1% formic acid) and analysed by LC-HRMS.

### LC-HRMS analysis

Analysis was conducted by a Q-Exactive Focus Hybrid-Quadrupol-Orbitrap analyser coupled to a Dionex UltiMate 3000 UHPLC chromatographic system (Thermo Fisher Scientific, Dreieich, Germany). Chromatographic separation was performed using gradient elution on a Nucleoshell RP 18 plus column (100 mm x 2.1 mm; 2.7 μm; Macherey-Nagel, Dueren, Germany) with a run time of 10 min as follows: 0.00–0.50 min 98% MP A at 0.5 mL/min, 0.50–1.00 min to 95% MP A at 0.5 mL/min, 1.00–3.00 min to 90% MP A at 0.5 mL/min, 3.00–3.50 min to 85% MP A at 0.5 mL/min, 3.50–4.00 min to 75% MP A at 0.5 mL/min, 4.00–5.75 min to 70% MP A at 0.5 mL/min, 5.75–6.50 min to 65% MP A at 0.5 mL/min, 6.50–7.40 min to 50% MP A at 0.5 mL/min, 7.40–7.85 min to 0% MP A to 0.75 mL/min, 7.85–8.60 min 0% MP A at 0.75 mL/min, 8.60–9.00 min to 98% MP A to 0.6 mL/min, 9.00–10.00 min 98% MP A to 0.5 mL/min [44]. The parameters for the washing steps were as follows: draw speed 4 µL/s with draw delay 0 ms, dispense speed 5 µL/s with dispense delay 0 ms, dispense to waste speed 5 µL/s. Washing was performed after draw with as wash volume of 50 µL of 1:10 *v:v* MeOH:Water (LC-MS-grade) and a wash speed of 4 µL/s. Total turnaround time was 12 min per sample. The column oven was set to a temperature of 35°C. The injection volume was 10 µL, the auto sampler temperature was 15°C. All samples were injected within one analytic batch sorted by storage condition (first conical centrifuge tube storage, afterwards petri dish storage) and within the storage condition ascending by incubation day. The pooled vitreous humour sample (control) was injected before, in-between and after the samples, which proved stable measurement of the instrument. Blanks (ACN:MeOH; 4:1; *v: v*) were injected before and after the samples as well as between the two storage conditions.

Ionisation was performed with heated-electrospray ionisation (HESI) in positive ionisation mode. The HRMS-source parameters were set to: spray voltage: 3500 V, capillary temperature: 320°C, sheath gas flow rate: 50 arbitrary units (AU), auxiliary (aux) gas flow rate: 20 AU, *S*-lens RF level: 55, probe heater temperature: 350°C. The HR mass spectrometer operated in full scan (FS) mode for analyte detection and peak area acquiring, while parallel reaction monitoring (PRM) was used for analyte identification. HRMS was performed with a resolution of 17,500. For the FS mode the scan range was *m/z* 101–600, for the PRM mode the normalised collision energy (NCE) was 22% and the isolation width *m/z* 1.1.

### Data interpretation

Twenty-one potential biochemical markers, including six AA (Lys, His, Arg, Tyr, Phe, and Trp) and 15 AAM (*N*^2^-acetyllysine (N2ALys), *N*^2^-acetylarginine (N2AArg), N6ALys, *N*-acetylcadaverine (NACad), *N*-acetylagmatine (NAAgm), Trm, *N*^2,6^-diacetyllysine (N2,6ALys), PEA, Kynurenic acid (KynA), *N*-acetyltyrosine (NATyr), NATrm, *N*-acetylphenylalanine (NAPhe), NATrp, NAPEA, *N*-acetyltryptophan (NATrp)) were monitored by LC-HRMS in (putrefied) vitreous humour samples over the experiment period. Starting with the beginning of the experiment (d0) the initial peak area of each analyte was determined and compared to the following days (d1, d2, d3, d4). Another comparison was drawn among the compound classes and the different storage conditions (conical centrifuge tube and petri dishes).

### Software and statistics

For LC-HRMS raw data analysis, the software XCalibur (Thermo Fisher Scientific) was used. Careful HRMS^2^ spectra evaluation and the software Sirius, including the tools CSI: FingerID, Structure Database Search (all DBs), and CANOPUS analysis (adduct: [M + H]^+^, instrument: orbitrap, MS^2^ mass accuracy: 10 ppm, MS/MS isotope scorer: ignore) were initially used to assign structures to analyte fragment spectra before analytical standards were purchased for confirmation [45–51]. Statistical analysis was performed in OriginPro 2024. Unpaired, two-tailed t-tests were applied with a significance level of α = 0.05. Obtained peak areas of each day and each condition were compared for each analyte. ChemDraw 22.0.0 was used to draw chemical structures.

## Results

Pig eyes, stored under controlled conditions within a climate chamber for up to four days, were macroscopically investigated and vitreous humour was analysed by LC-HRMS for changes in AA profiles and microbial formation of AAM/degradation products (Fig. 1).

### Macroscopic changes of *postmortem* pig eyes over time

At the beginning of the examination, the pig eyes showed the typical rounded shape with elastic consistency and a shiny, slightly moist surface (Online Resource 1 – Fig. 1). The corneas were clear, the sclera predominantly white with outlying gray pigment spots. Depending on the size of the eyes, between 2 mL and 3.5 mL of clear liquid containing highly viscous streaks could be aspirated from the vitreous body. No difference was observed between eyes on Petri dishes and in conical centrifuge tubes. After 24 h, the corneas started to become turbid and the surfaces began to dry out. The reverse side of the eyes on Petri dishes began to turn dark in the former white parts of the corneas. The reverse sides of the eyes in the conical centrifuge tubes dried out considerably less and there was no discolouration. In the following days, the surfaces increasingly desiccated and the corneas became turbid. The amount of fluid obtained through puncture decreased with longer incubation times and the fluid became more viscous, cloudier and was increasingly interspersed with dark gray particles. In some cases, it was no longer possible to obtain vitreous humour at all due to increasing dehydration. Concerning the eyes on Petri dishes, in particular reversed eye sides desiccated and became darker in colour turning almost black over time. The eyes shrank in height and, to a lesser extent, in diameter, resulting in a discus-like shape. The surfaces of the eyes in conical centrifuge tubes desiccated less severely, in particular reversed sides became moister and small amounts of a pink, slightly cloudy liquid accumulated at the bases of the conical centrifuge tubes. The parts of the sclera exposed to the surrounding air became yellowish and greasy without darkening. The eyes lost height while the diameter remained approximately the same.

### Changes of peak areas of amino acids and their metabolites/degradation products over time

LC-HRMS analysis of AA and AAM was performed to evaluate their potential as biochemical markers for PM alterations. Based on the previously described NATrm (a Tyr metabolite) further AAM were anticipated to be contained in putrefied body fluids, such as putrefied vitreous humour. These anticipated substances could be putatively identified in HRMS FS data of the vitreous humour samples based on the accurate masses of their protonated molecules and additional careful interpretation of the obtained HRMS^2^ spectra with the support of in silico prediction. Analytical standards were purchased or else self-produced for confirmation or rejection of the putatively assigned analytes to HRMS^2^-spectra. In total 18 analytical standards for AA and AAM (Lys, His, Cad, Arg, Agm, Htm, Oct, N6ALys, NAHtm, Tyr, Trm, Phe, PEA, Trp, Try, NATrm, NAPEA, and NATry) were purchased. Nine AAM were self-produced by acetylation (N2ALys, *N*-acetylhistidine (NAHis), N2AArg, NACad, NAAgm, N2,6ALys, NATyr, NAPhe, NATrp), KynA had been identified previously. Thus, HRMS^2^-spectra were obtained for 28 AA/AAM, details are given in Table 1.

**Table 1:** Novel potential biochemical markers for putrefaction. Given are tested amino acids and metabolites/degradation products sorted by retention time t_R_ with their abbreviations, molecular formulas, molecular ion peaks, specific HRMS^2^ fragments (NCE = 22%) as well as detection in vitreous humour samples

t_*R*_ [min]	Biochemical Marker	Abbreviation	Molecular Formula	[M + H]^+ ^Exact Mass [Da]	HRMS^2^ Fragments Accurate Mass [Da]	Detected in samples
0.87	Lysine	Lys	C_6_H_14_N_2_O_2_	147.11280	84.08133, 130.08621	Yes
0.89	Histidine	His	C_6_H_9_N_3_O_2_	156.07675	95.06081, 110.07137	Yes
0.91	Cadaverine	Cad	C_5_H_14_N_2_	103.12298	86.09705	No
0.91	Arginine	Arg	C_6_H_14_N_4_O_2_	175.11895	60.05641, 70.06580	Yes
1.00	Agmatine	Agm	C_5_H_14_N_4_	131.12912	60.05638, 72.08140	No
1.02	*N*^*2*^-Acetyllysine	N2ALys	C_8_H_16_N_2_O_3_	189.12337	84.08140, 129.10225	Yes
1.02	*N*-Acetylhistidine	NAHis	C_8_H_11_N_3_O_3_	198.08732	110.07160, 156.07681	No
1.10	Histamine	Htm	C_5_H_9_N_3_	112.08692	95.06075	No
1.15	Octopamine	Oct	C_8_H_11_NO_2_	154.08626	136.07559	No
1.16	*N*^*2*^-Acetylarginine	N2AArg	C_8_H_16_N_4_O_3_	217.12952	70.06583, 84.08133	Yes
1.17	*N*^*6*^-Acetyllysine	N6ALys	C_8_H_16_N_2_O_3_	189.12337	84.08140, 126.09148	Yes
1.25	*N*-Acetylhistamine	NAHtm	C_7_H_11_N_3_O	154.09749	95.06076, 112.08704	No
1.42	*N*-Acetylcadaverine	NACad	C_7_H_16_N_2_O	145.13354	86.09704, 128.10716	Yes
1.63	Tyrosine	Tyr	C_9_H_11_NO_3_	182.08117	136.07578, 165.05479	Yes
1.86	*N*-Acetylagmatine	NAAgm	C_7_H_16_N_4_O	173.13969	114.09160, 156.11307	Yes
2.30	Tyramine	Trm	C_8_H_11_NO	138.09134	121.06482	Yes
2.80	Phenylalanine	Phe	C_9_H_11_NO_2_	166.08626	120.08083	Yes
2.97	*N*^*2,6*^-Diacetyllysine	N2,6ALys	C_10_H_18_N_2_O_4_	231.13393	84.08127, 126.09158	Yes
3.85	2-Phenylethylamine	PEA	C_8_H_11_N	122.09643	105.07011	Yes
3.91	Tryptophan	Trp	C_11_H_12_N_2_O_2_	205.09715	146.06001, 188.07054	Yes
4.12	Kynurenic acid	KynA	C_10_H_7_NO_3_	190.04987	162.05495	Yes
4.21	*N*-Acetyltyrosine	NATyr	C_11_H_13_NO_4_	224.09173	136.07541, 182.08131	Yes
5.00	Tryptamine	Try	C_10_H_12_N_2_	161.10732	144.08052	No
5.14	*N*-Acetyltyramine	NATrm	C_10_H_13_NO_2_	180.10191	121.06495, 138.09134	Yes
5.69	*N*-Acetylphenylalanine	NAPhe	C_11_H_13_NO_3_	208.09682	120.08100, 166.08624	Yes
5.91	*N*-Acetyltryptophan	NATrp	C_13_H_14_N_2_O_3_	247.10772	188.07060, 201.10220	Yes
6.45	*N*-Acetyl-2-phenylethylamine	NAPEA	C_10_H_13_NO	164.10699	105.07018, 122.09657	Yes
6.73	*N*-Acetyltryptamine	NATry	C_12_H_14_N_2_O	203.11789	144.08052	Yes

**Fig. 2:**
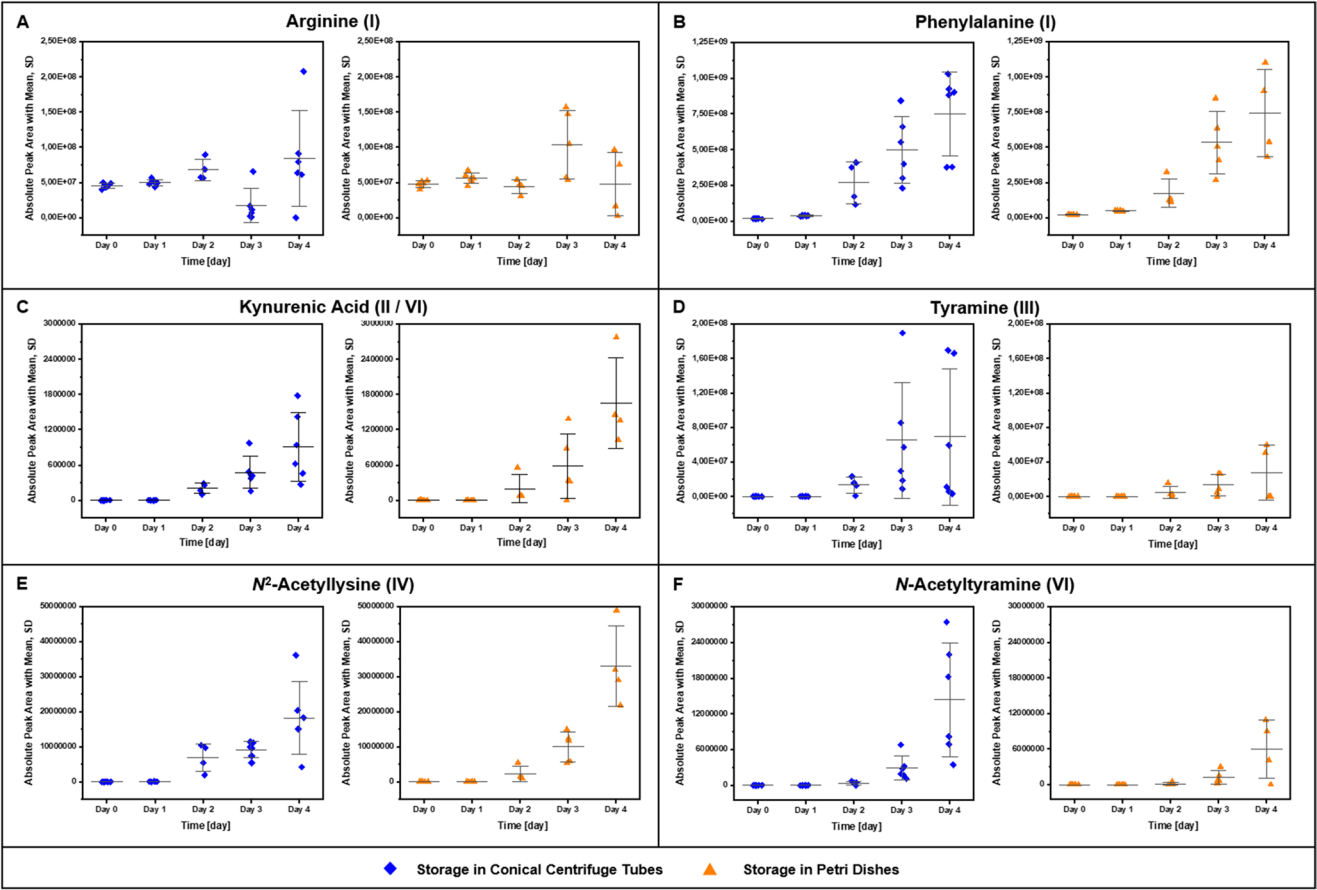
Detected absolute peak areas of selected analytes. Absolute peak areas are shown for both storage conditions in conical centrifuge tubes and petri dishes for the four experiment days with A– arginine (amino acid, (I)), B– phenylalanine (amino acid, (I)), C– kynurenic acid (transamination product of the tryptophan metabolite kynurenine, (II, VI)), D– tyramine (biogenic amine of tyrosine, (III)), E– N2-acetyllysine (N-acetylated product of lysine, (V)), F– N-acetyltyramine (N-acetylated product of tyramine, (VI))

In vitreous humour from pig eyes, 21 of these AA/AAM, namely Lys, His, Arg, N2ALys, N2AArg, N6ALys, NACad, Tyr, NAAgm, Trm, Phe, N2,6ALys, PEA, Trp, KynA, NATyr, NATrm, NAPhe, NATrp, NAPEA, and NATry, were detected during the experiment (see Table 1). Online Resource 1– Fig. 2 shows the chromatographic separation of these 21 analytes.

The 21 potential biochemical markers were monitored in putrefying vitreous humour from pig eyes stored on either conical centrifuge tubes or Petri dishes over four days. The absolute peak areas of selected analytes are depicted in Fig. 2.

**Fig. 3:**
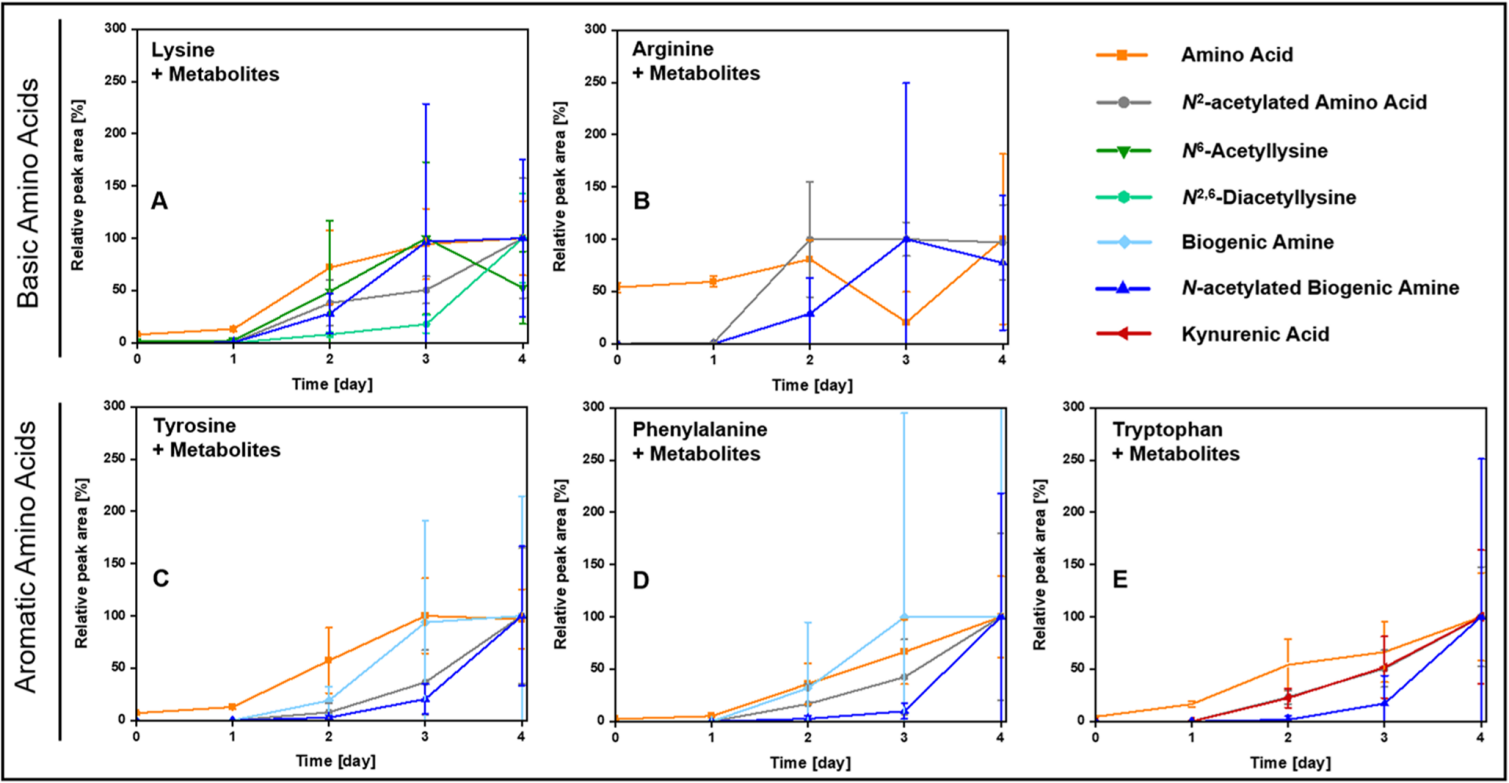
Relative peak areas of the five investigated amino acids with their respective metabolites/degradation products. Exemplarily shown for the conical centrifuge storage condition. Relative peak areas are given in % as normalized values against the highest peak area mean value of each analyte over four days. A– lysine with *N*^2^-acetyllysine, *N*^6^-acetyllysine, *N*^2,6^-acetyllysine, *N*-acetylcadaverine; B– arginine with *N*^2^-acetylarginine and *N*-acetylagmatine; C– tyrosine with *N*-acetyltyrosine, tyramine, *N*-acetyltyramine; D– phenylalanine with *N*-acetylphenylalanine, 2-phenylethylamine, *N*-acetyl-2-phenylethylamine; E– tryptophan with *N*-acetyltryptophan, *N*-acetyltryptamine, kynurenic acid

The respective information of all analytes may be found in Online Resource 1– Fig. 3 as well as details in Online Resource 2– Table 1.

All of the investigated AA and AAM, with exception of Arg (Fig. 2A), had in common, that their peak areas increased with progressing time as exemplarily shown for Phe, KynA, Trm, N2ALys, and NATrm in Fig. 2B-F. The six substrate AA (I) Lys, His, Arg, Tyr, Phe, and Trp and *N*-monoacetylated Lys (N2ALys, N6ALys) were already present at d0. Lys, Tyr, Phe, and Trp had a flat slope until d1 and afterwards their peak areas increased rapidly by up to 46-fold (Phe, see Fig. 2B). For Lys (Online Resource 1– Fig. 3A) and Tyr (Online Resource 1– Fig. 3C), a plateau phase was reached at d3 (sigmoidal curve appearance), His peak areas decreased after d3 (Online Resource 2– Table 1). Arg (Fig. 2A) was the only AA for which no trend with respect to peak areas could be observed over time. As no His metabolites/degradation products were found, His was not further investigated in this study.

In contrast to N2ALys (Fig. 2E), N2AArg, and N6ALys, the other 12 AAM (NACad, NAAgm, Trm (Fig. 2D), N2,6ALys, PEA, KynA (Fig. 2C), NATyr, NATrm, NAPhe, NATrp, NAPEA, NATry) were not detected or only as traces at the start (d0) of the experiment and the first day (d1) (Online Resource 1– Fig. 3, Online Resource 2– Table 1). Detection of AAM/degradation products began most frequently at the second day (d2) (NACad, NAAgm, Trm, N2,6ALys, PEA, KynA, NATrm, NAPhe, NATrp, NAPEA) or the third day (d3; NATyr, NATry) of the experiment. Most AAM showed a similar development of peak areas over time with a moderate slope until d3 and a strongly increased slope from d3 to d4.

Particularly, for the AAM KynA (Fig. 2C), N2ALys (Fig. 2E), NATrm (Fig. 2F), and NATrp significant increases over time were observed for both storage conditions (t-test, α = 0.05 for comparison of each day to every other single day, Online Resource 2– Tables 2 and 3). Partially, high standard deviations (Online Resource 2– Table 1) were found for the measured absolute peak areas. Thus, ratios between all AAM and respective AA were calculated. Additionally, ratios between acetylated biogenic amines and their respective biogenic amine and acetylated AA were calculated. An increase of the above mentioned ratios was observed over time as for the absolute peak areas. Standard deviations could not be reduced by this approach.

Fig. 3 shows the peak area development for the AA with their respective AAM of the conical centrifuge storage condition in one graph given as relative peak area in % with the highest peak area mean value of each analyte set equal to 100%.

Following increased peak areas of the substrate AA (I), its respective AAM (II–VI) peak areas increased with delay. This was especially true for the Tyr signal (relative peak area in %), which was followed by the Trm signal and afterwards the NATrm signal (Fig. 3C). Similar results were found for Lys, Phe, and Trp. For KynA and NATrp a very similar peak area development was observed (Fig. 3E, Online Resource 2– Table 1).

Partially, large variation (%RSD) was found among the six pig eyes of both conditions, particularly for the longer storage time points d2-d4 and the analytes NAAgm (172%, d2 petri dish), Trm (141%, d2 petri dish, Fig. 2D), PEA (216%, d3 petri dish), and NAPEA (181%, d2 petri dish). Lowest variation was observed for Lys on d1 (7%, petri dish). With respect to the two storage conditions either on truncated conical centrifuge tubes or petri dishes (Fig. 2, Online Resource 1– Fig. 3, Online Resource 2– Table 1), only few significant differences between mean peak areas of the investigated analytes were observed (t-test, α = 0.05): Arg, slightly higher in conical centrifuge tubes on day 2 and markedly higher in petri dishes on day 3 (Fig. 2A); N2AArg, slightly higher in petri dishes on day 4; N6ALys, markedly higher in conical centrifuge tubes on day 3; NATyr, slightly higher in petri dishes on day 4; and NAPhe, markedly higher in petri dishes on day 4 (Online Resource 1– Fig. 3).

## Discussion

The intact eye surface is inhabited by microorganisms (MO; ocular flora) that interact with the host’s immune system and restrict the growth of single MO populations to prevent infections [52]. The interior of the eye is normally protected from infections by bacteria, viruses, and fungi due to the eye’s anatomic isolation (e.g., sclera, cornea, blood-retinal-barrier) [1, 30, 39–41, 52, 53] unless the eye is injured e.g., by trauma [52]. PM, however, sclera and cornea become desiccated, cell membranes degrade and hydrolytic enzymes are released (autolysis) making these protective structures permeable for invading bacteria (putrefaction) [4, 54].

In this study, pig eyes were incubated in a climate chamber on either conical centrifuge tubes or petri dishes at 25°C and 65% humidity and observed for macroscopic changes and changes of metabolic patterns in vitreous humour over four days. The first macroscopic changes were (mild) opacification of corneas and dry surfaces seen within 24 h (Online Resource 1– Fig. 1), which matches with descriptions given in the literature [28, 42, 43, 55]. In an intact eye, corneal transparency is assured by the endothelium preventing stromal water uptake. In an autolytic eye, however, endothelium degrades and is no longer able to hinder the aqueous humour from hydrating the cornea which leads to the hazy appearance [56]. Cantürk et al*.* took pictures of eyes from ten individuals every 20 min for 15 h and used computer-based techniques to analyse corneal and non-corneal opacities. They found a significant correlation between time since death and opacity [55]. Nevertheless, this opacification process was seen to be influenced by temperature and humidity [28, 42] and other factors such as age and eye colour might have an influence too. Opacity became stronger in the investigated pig eyes with time as well and further observations included shrinkage of the eyes, especially in height, which was more pronounced for eyes on petri dishes compared to conical centrifuge tubes. This might be explained by the increased air exposure and thus desiccation of eyes on petri dishes. Whereas in truncated conical centrifuge tubes the eyes air exposure was more similar to the orbital cavity, which could also explain the differences in discolouration between eyes on petri dishes (dark reverse sides) and eyes on conical centrifuge tubes (no discolouration at reverse sides, slightly yellowish colour of air exposed sclera) probably in combination with bacterial activity. Overall, macroscopic changes of eyes in conical centrifuge tubes appeared more comparable to detectable changes in eyes from autopsy specimens than changes in eyes on petri dishes.

Due to its isolated anatomy, PM biochemical changes are believed to proceed slower within the eye. Thus, ocular fluids have an advantage over other body fluids with respect to PMI investigations [1, 14, 30–33, 39–43]. In this study, every day of the experiment vitreous humour was drawn from up to six eyes of each storage condition. With progressing time the obtained amount of vitreous fluid decreased and it became more viscous and dark in accordance with superficially observed changes. For later time points (d2-4) it was sometimes not possible to obtain vitreous humour from all six eyes due to desiccation (4 ≤ *n* ≤ 6). LC-HRMS analysis was performed with the vitreous humour samples to investigate a possible formation or degradation of AA and AAM (Fig. 1). AA and AAM are present in physiologic biological samples, thus they are already subject of research for different forensic applications including PMI estimation [44, 57–60].

With respect to the proteolysis end products (I) AA (Fig. 1), basic (Lys, His, Arg) and aromatic (Phe, Tyr, Trp) AA were the focus of this study. These AA were detected in all investigated vitreous humour samples and their peak areas increased over time, which was significant for all AA except for Arg (Fig. 2A), where no trend was observed (Online Resource 1– Fig. 3, Online Resource 2– Tables 1, 2 and 3). While others claimed a linear or logarithmic linear correlation ship between AA abundance and PMI [36, 61], the LC-HRMS detected peak areas of AA determined in this study increased either in a (slightly) sigmoidal way (Lys, Tyr, Phe; Fig. 2B, Online Resource 1– Fig. 3A, C, D), as a double-S curve (Trp, Online Resource 1– Fig. 3E) or no correlation was found (Arg). Nevertheless, as the variation among the eyes from the same experimental day and storage condition was high, especially at later time points, and the sample size was low (4 ≤ *n* ≤ 6) one should avoid over-interpretation of these curve shapes. Additionally, while Girela et al*.* found significantly increased AA/AAM concentrations (aspartate, glutamate, taurine), a linear correlation between AA concentration and PMI must not implicitly be the conclusion indicated by correlation-coefficients (0.3191 ≤ *r* ≤ 0.4508) obtained in their study [36, 59]. In an early study from 1976, Bonte et al*.* described protein and AA degradation in putrefied fluid from the thoracic cavity of dogs. They found a biphasic AA concentration development (including Lys, Tyr, Phe) with two incline and following decline phases over a period of 45 days at 10°C [62].

In the putrefaction process, AA may be metabolised/degraded further to different types of AAM as a result of (II) deamination/transamination, (III) decarboxylation, (IV) hydroxylation, and (V) *N*-acetylation (Fig. 1) [21]. For the observed AAM, most probably formation was observed as they were not present or only in traces (N2ALys, L6ALys) at d0 and increased until d4, which could indicate bacterial metabolism following MO invasion of the normally protected interior of the eye [1, 30, 39–41, 52]. 

With respect to (II) deamination/transamination (Fig. 1), only KynA, a Trp metabolite formed from the AA kynurenine by the action of kynurenine aminotransferase, was found [63]. The results indicate, that KynA is initially not present in vitreous humour, whereas it is present e.g., in other body fluids such as cerebrospinal fluid and urine [64]. In a previous study, KynA was investigated amongst other endogenous biomolecules in dryed urine spots over two months [44]. In this setting, KynA was found to be rather stable unless exposed to sunlight or high humidity. For the high humidity condition, two fungi species were identified (*Aspergillus versicolor* and *Penicillium chrysogenum*) which could have degraded KynA [44]. In contrast, KynA was first detected on d2 in the present study and peak areas increased until d4 (Fig. 2C) indicating presence of MO possessing kynurenine aminotransferase activity, such as *Escherichia coli* [65].

Another degradation pathway of AA is (III) decarboxylation (Fig. 1), which results in the formation of biogenic amines. Among the most prominent biogenic amines of putrefaction are Cad and putrescine causing the typical decomposition odour [19, 22–24]. The six corresponding biogenic amines to Lys, His, Arg, Tyr, Phe, and Trp; namely Cad, Htm, Agm, Trm, PEA, and Try were investigated (Online Resource 2– Table 1). There are specialised decarboxylases for basic AA, such as lysine decarboxylase expressed by enterobacteria to counteract acid stress, a condition bacteria experience in a host’s digestive tract [66], but also PM during autolysis/putrefaction [3, 13]. Aromatic amines are formed by the action of aromatic acid decarboxylases, enzymes expressed by gut bacteria. Sugiyama et al*.* identified five bacterial species (*Blautia hansenii*,* Clostridium asparagiforme*,* Tyzerella nexilis*,* Enterococcus faecalis*,* Ruminococcus gnavus*), which were able to produce Trm, PEA, and Try [67]. The composition of biogenic amines is highly variable and correlated to the present microbes [19]. Thus, *Sherwanella putrefaciens* was found to produce for example di/trimethylamine [68]. Bonte et al*.* performed two-dimensional immuno-electrophoresis with putrefied thoracic fluid samples and identified six biogenic amines, including Cad, Agm, PEA and Trm showing bell-shaped concentration curves with maxima between day 20 and 30 (at 10°C), whereas they did not find Try. Simultaneously, they counted bacterial growth, which was strongly increased for gram-positive rods between day 20 and 30 as well [62]. In the vitreous humour samples of the present study, two aromatic amines– Trm (Fig. 2D, Online Resource 1– Fig. 3C) and PEA (Online Resource 1– Fig. 3D)– were detected at d2 and their peak areas increased with their substrate AA over time as shown in Fig. 3C and D, which is a depiction of the mean (relative) peak area development of each AA with its detected AAM over time normalised to the highest mean peak area of each analyte and given in %. This agrees with the hypothesis of MO producing biogenic amines during putrefaction processes.

With respect to (IV) hydroxylation of AA (Fig. 1), only Tyr, which corresponds to hydroxylated Phe, was found (described above). Furthermore, a hydroxylated biogenic amine, Oct (hydroxylated Trm), was investigated but not detected in the vitreous fluid samples of this study.

Last but not least there is *N*-acetylation, which was found for both AA and biogenic amines. (V) *N*-acetylated AA (Fig. 1) are produced in eukaryotic cells either by the action of *N*-acetyltransferases or by degradation of *N*-acetylated proteins as many proteins are acetylated at their *N*-termini or Lys side chains (*N*^6^), e.g. activated histones [69, 70]. For the five AA Lys, Arg, Tyr, Phe, and Trp increasing peak areas of their *N*-acetylated metabolites N2ALys (Fig. 2E), N6ALys, N2AArg, NATyr, NAPhe, and NATrp (Online Resource 1– Fig. 3A-E) were found (with exception of N2AArg stored in conical centrifuge tubes). This is in accordance to a study where *N*-acetylalanine, *N*-acetylvaline, and *N*-acetylornithine were found to increase in fermenting shrimps [71]. For Lys, three *N*-acetylated AAM were found with N2ALys being acetylated on the α-amino group, N6ALys being acetylated on the ε-amino group (side chain) and N2,6ALys being acetylated on both amino groups. *N*-acetylation of Lys-residues in proteins is a frequently observed mechanism in bacteria and was found to be increased with glycolysis as Acetyl phosphate is able to non-enzymatically acetylate Lys residues [72].

(VI) Bacteria metabolise mono- and polyamines (Fig. 1) under low nitrogen conditions, under cell stress (e.g. acidic conditions), and also to get rid of these compounds if they are available in excess [73]. Polyamine assimilation in bacteria involves different pathways including the ‘acetylation pathway’, which was found for spermine and spermidine in different bacteria [73]. Forouhar et al*.* identified and investigated the *N*-spermidine/spermine acetyltransferase PaiA and found a similar substrate activity of spermidine and Agm, even if Agm seemed to be too small to fit in the substrate binding site [74]. The biological role and further metabolism of acetylated polyamines, however, is not well studied yet [73]. With respect to NACad, diaminopentane acetyltransferase, an enzyme capable to acetylate Cad, was found in *Corynebacterium glutamicum*, its physiological role is so far unknown [75]. With respect to acetylation of aromatic biogenic amines (arylalkylamines), arylalkylamine *N*-acetyltransferases (Eis proteins) were identified in different Gram-positive bacteria, using as substrates e.g., Trm, Oct, PEA, and Try [76]. In this study, acetylated products were found for all five investigated biogenic amines starting at d2 and increasing over time as exemplified for NATrm in Fig. 2F (others are shown in Online Resource 1– Fig. 3A, B, D, E) with the increases lagging behind those of the respective biogenic amines (Fig. 3C, D). Thus, microbial formation in the course of putrefaction seemed likely.

With respect to variation among AA/AAM peak areas of the six eyes of each experiment day, there were in part markedly different peak areas observed (Online Resource 1– Fig. 3, Online Resource 2– Table 1), which indicates that there is large inter-individual variation even though all eyes were stored under the exact same condition at 25°C and 65% humidity in a climate chamber. This complicates the determination of the putrefaction grade and is a factor that has to be addressed further. Nevertheless, biogenic amines, acetylated AA (except N2ALys, N6ALys), and acetylated biogenic amines were not present in freshly drawn vitreous humour, but were detected from the second day (d2) of the experiment, which allows the prediction, that, if these AA/AAM are present, eyes stored under the given condition have been in the process of decomposition for at least two days. With respect to variation between the different storage conditions (conical centrifuge tubes or petri dishes), there were only few significant differences observed (Online Resource 1– Fig. 3). These two storage conditions were chosen to figure out if they have an influence on both macroscopic and metabolic changes. As discussed above, it had an influence on macroscopic changes due to differences in desiccation. With respect to AA and AAM formation differences between the storage conditions were not significant for most analytes in this study. For Arg and N2AArg there were significant differences observed, but Arg and its metabolites showed more inconsistent results in general, for which no explanation was found so far. It could be speculated that Arg was used in other microbial metabolic pathways co-occurring and not investigated in this study.

With respect to the characterisation of the putrefaction process, promising results were found for (I) AA: Lys, Tyr, Phe, Trp, for (II) the transamination product: KynA, as well as for the (V) *N*-acetylated AA N2ALys, NATrp and (VI) *N*-acetylated biogenic amines (Fig. 1), especially NATrm.

## Conclusion

An estimation of the PM interval based on PM metabolic changes is difficult as these changes depend on many environmental factors. Thus, the aim of this study was to characterise the putrefaction grade by potential putrefaction markers putatively formed by MO invading the (in vivo) more or less sterile interior of the eye. Overall AAM, especially the Trp metabolite KynA but also the *N*-acetylated AAM N2ALys, NATrp and the acetylated biogenic amine NATrm, seemed to be promising targets as potential putrefaction markers, especially if evaluated in combination with macroscopic changes such as opacity. Nevertheless, large variation in AA/AAM peak areas was observed suggesting strong inter-individual differences in metabolic profiles. Thus, further studies are needed for evaluation of inter-individual but also intra-individual (two eyes of one individual) variations, the microbial burden, a (if possible) longer monitoring time, and AA/AAM formation in a more realistic scenario such as the influence of different environmental conditions and investigation of eyes in their normal anatomic location– the orbital cavity of a cadaver.

## Supplementary Information

Below is the link to the electronic supplementary material.

ESM 1 (DOCX 1.22 MB)

ESM 2 (DOCX 54.1 KB)

## Data Availability

The datasets generated during and/or analysed during the current study are available from the corresponding author on reasonable request.
