# Supplementary material for: Amino acids and their metabolites as potential biochemical markers in postmortem vitreous humour
Source: Int J Legal Med. 2025 Jul 2;139(6):3051–62. doi: 10.1007/s00414-025-03552-9 (PMC12672791; doi:10.1007/s00414-025-03552-9)
Supplement: Supplementary file 1 — (DOCX 1.22 MB) [file 414_2025_3552_MOESM1_ESM.docx]

**Online Resource 1**

International Journal of Legal Medicine

**Amino acids and their Metabolites as potential biochemical Markers in *postmortem* Vitreous Humour**

Laura Franke^1^*, Hannah Ihle^1^*, Kristina Rieger^1^, Viviane Stammer^1^, Senta Niederegger^1^, Dirk K. Wissenbach^1^, Frank T. Peters^1^, Gita Mall^1^

^1^Jena University Hospital, Institute for Forensic Medicine, Friedrich Schiller University Jena, Germany

Author to whom correspondence should be addressed. Email: dirk.wissenbach@med.uni-jena.de

**
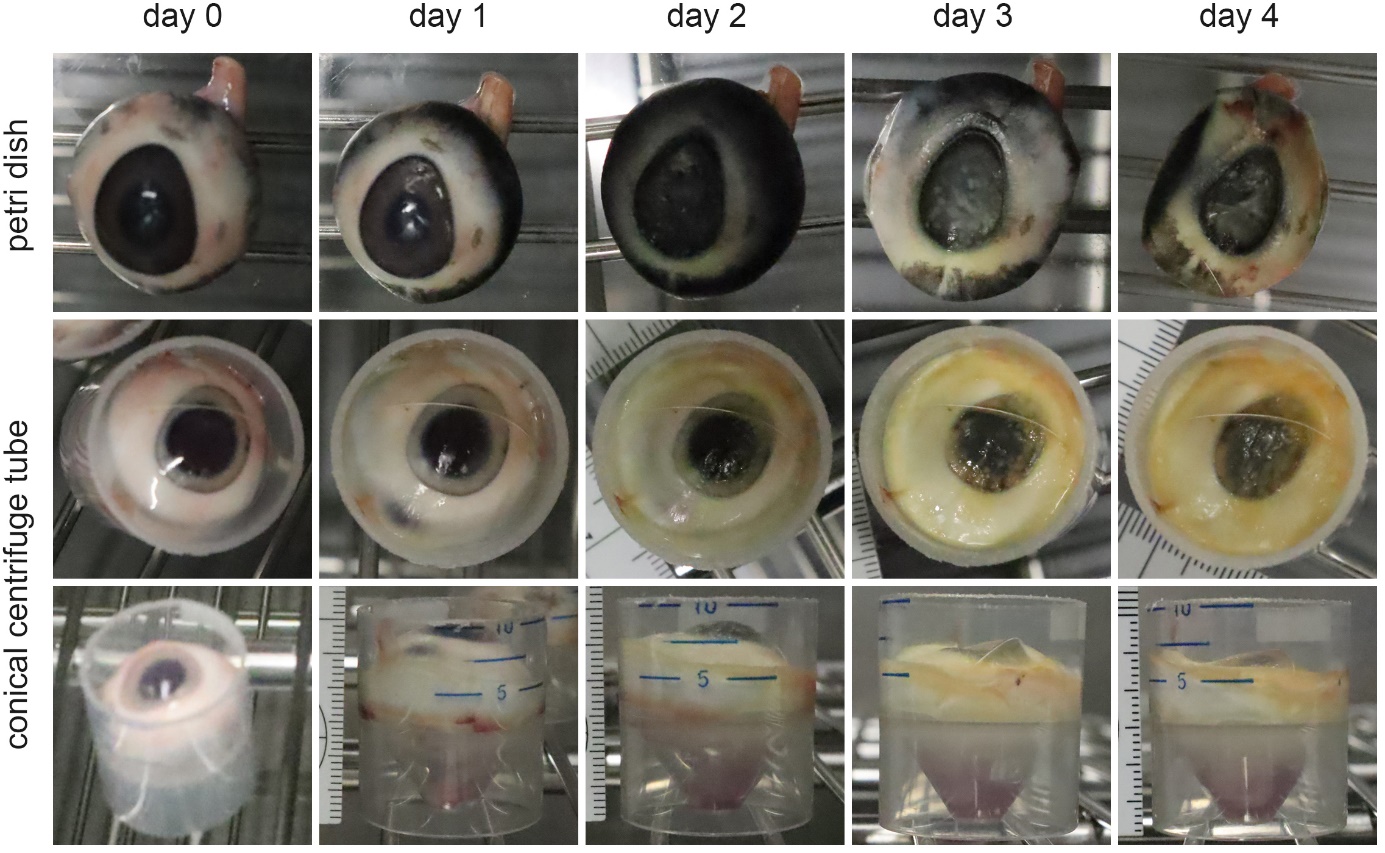
**

***Online Resource 1 – Fig. 1: Postmortem macroscopic changes of pig eyes.*** *Depicted are photographs from pig eyes stored in conical centrifuge tubes and petri dishes in a climate chamber at 25°C and 65% humidity for up to four days*


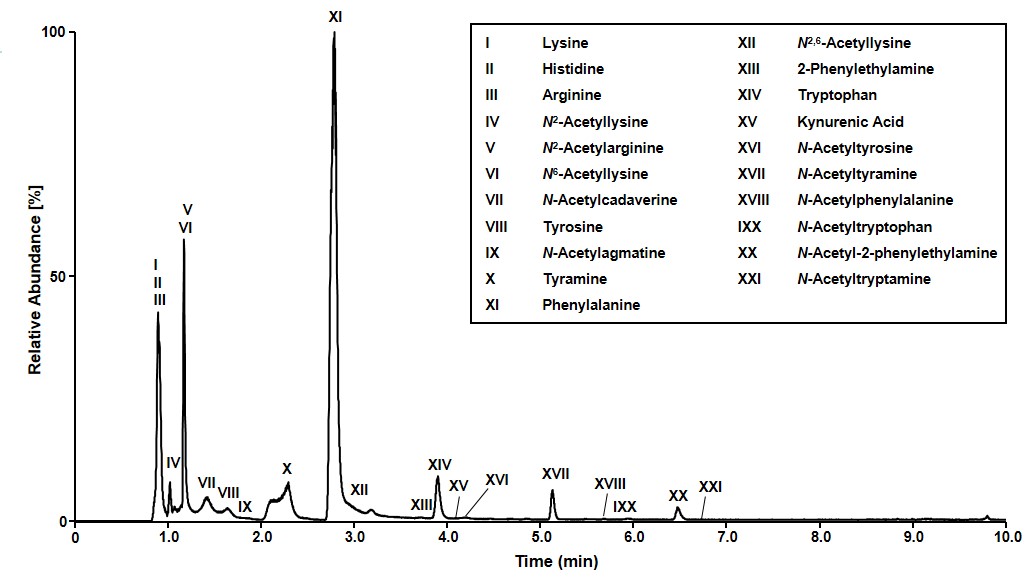


***Online Resource 1 – Fig. 2: Extracted ion chromatograms of 21 amino acids and amino acid metabolites.*** *Exemplarily shown for vitreous humour drawn from a pig’s eye, stored in a conical centrifuge tube for four days at 25°C, 65% humidity in a climate chamber*

**
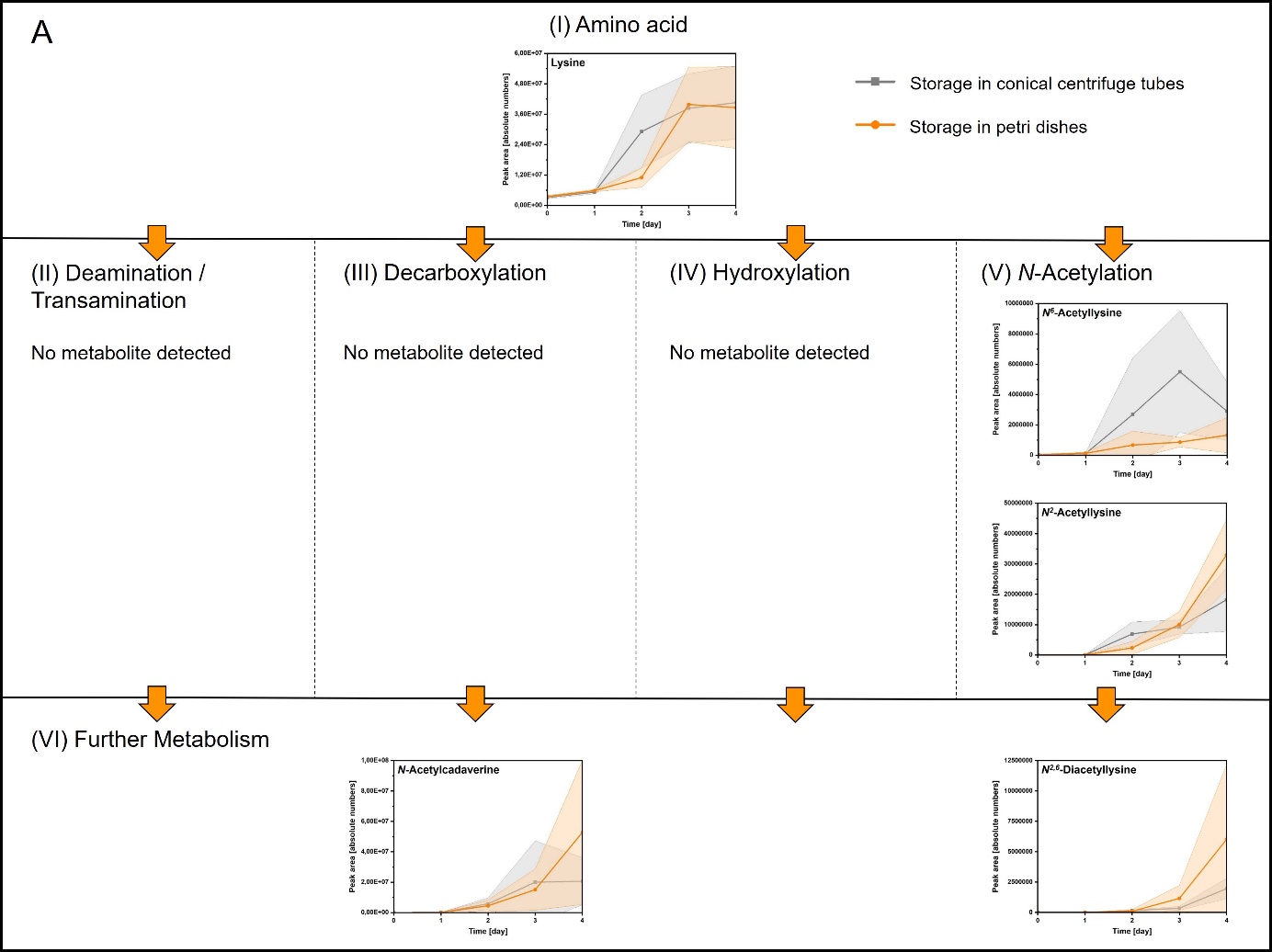
**

**
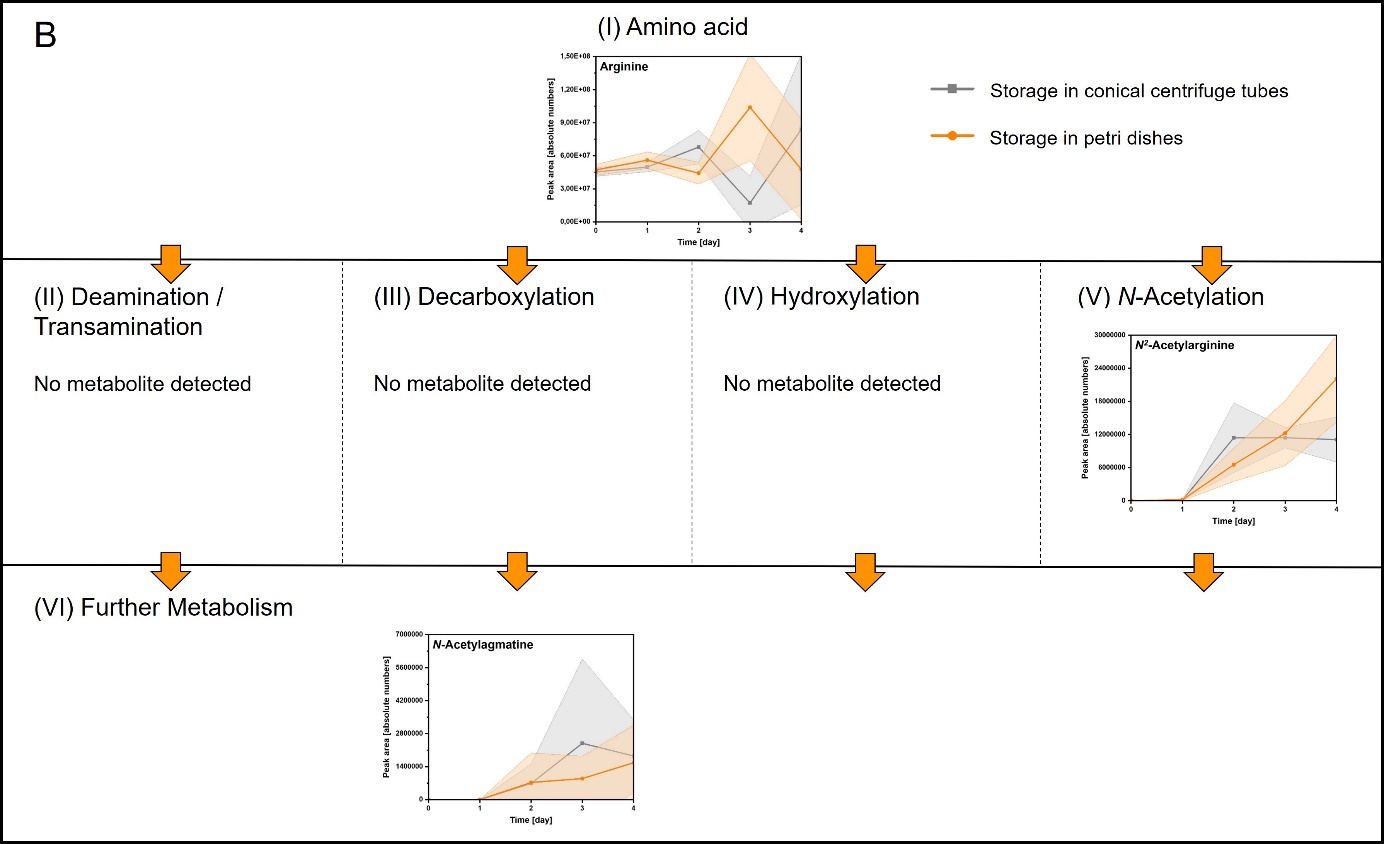
**

**
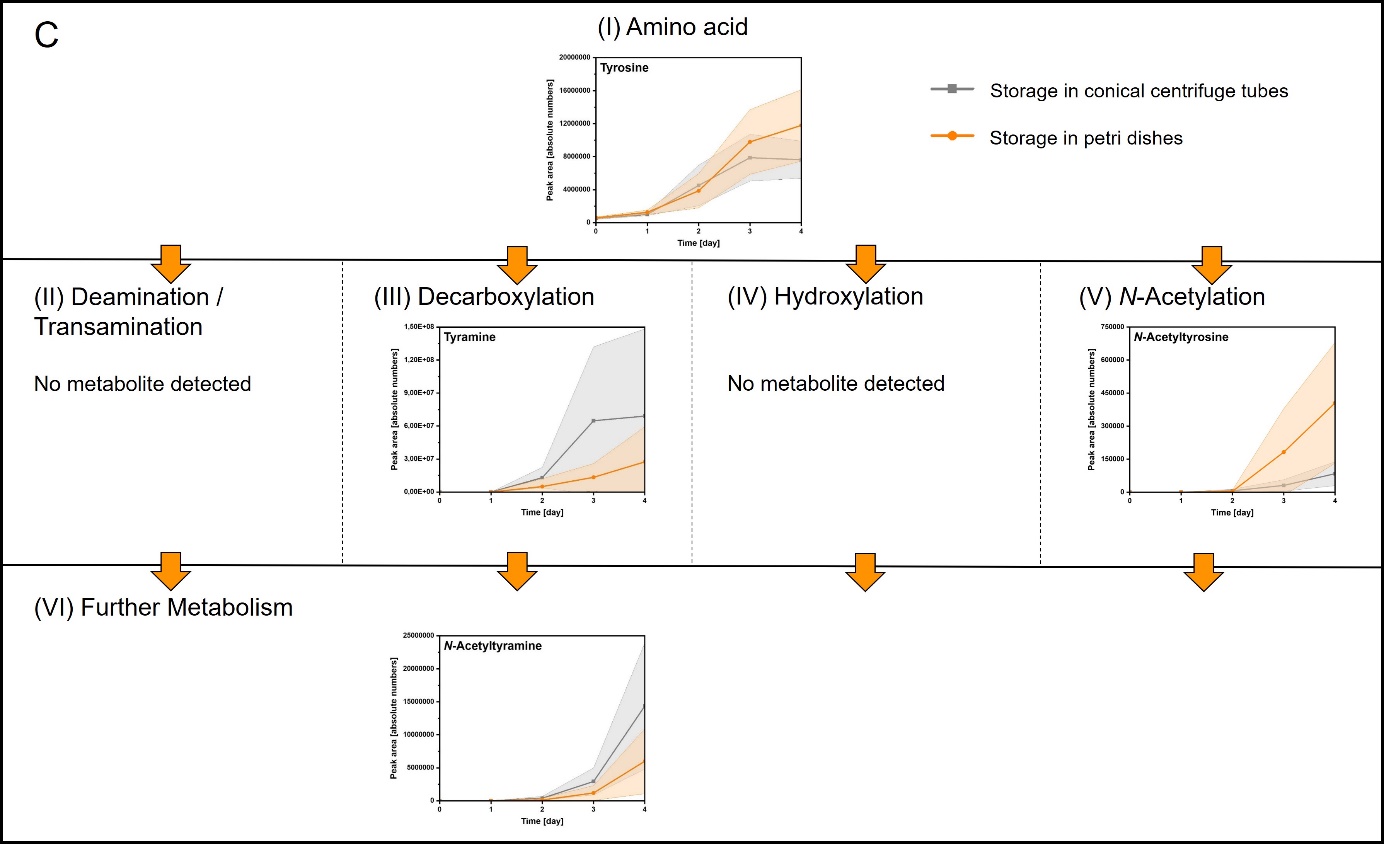
**

**
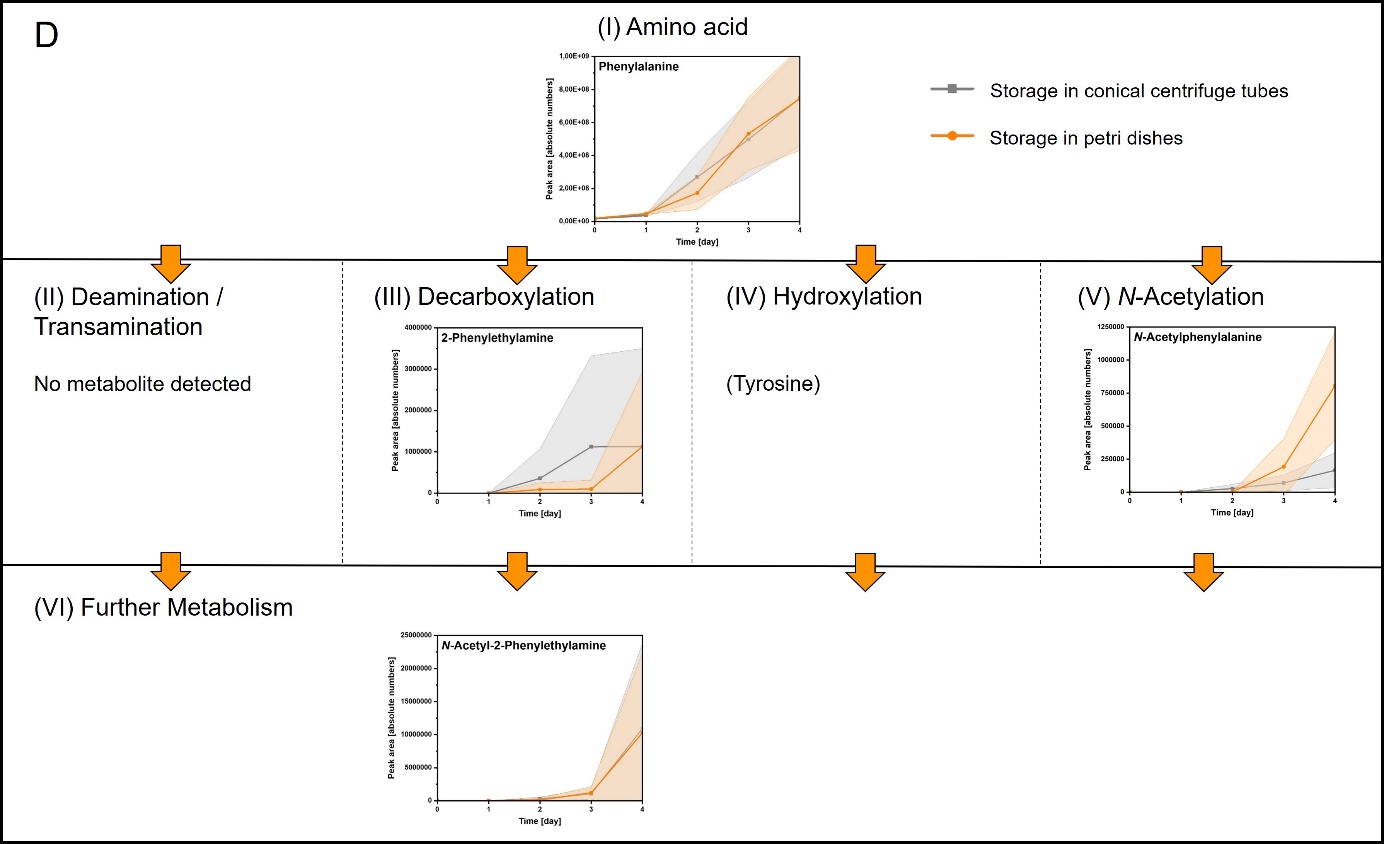
**

**
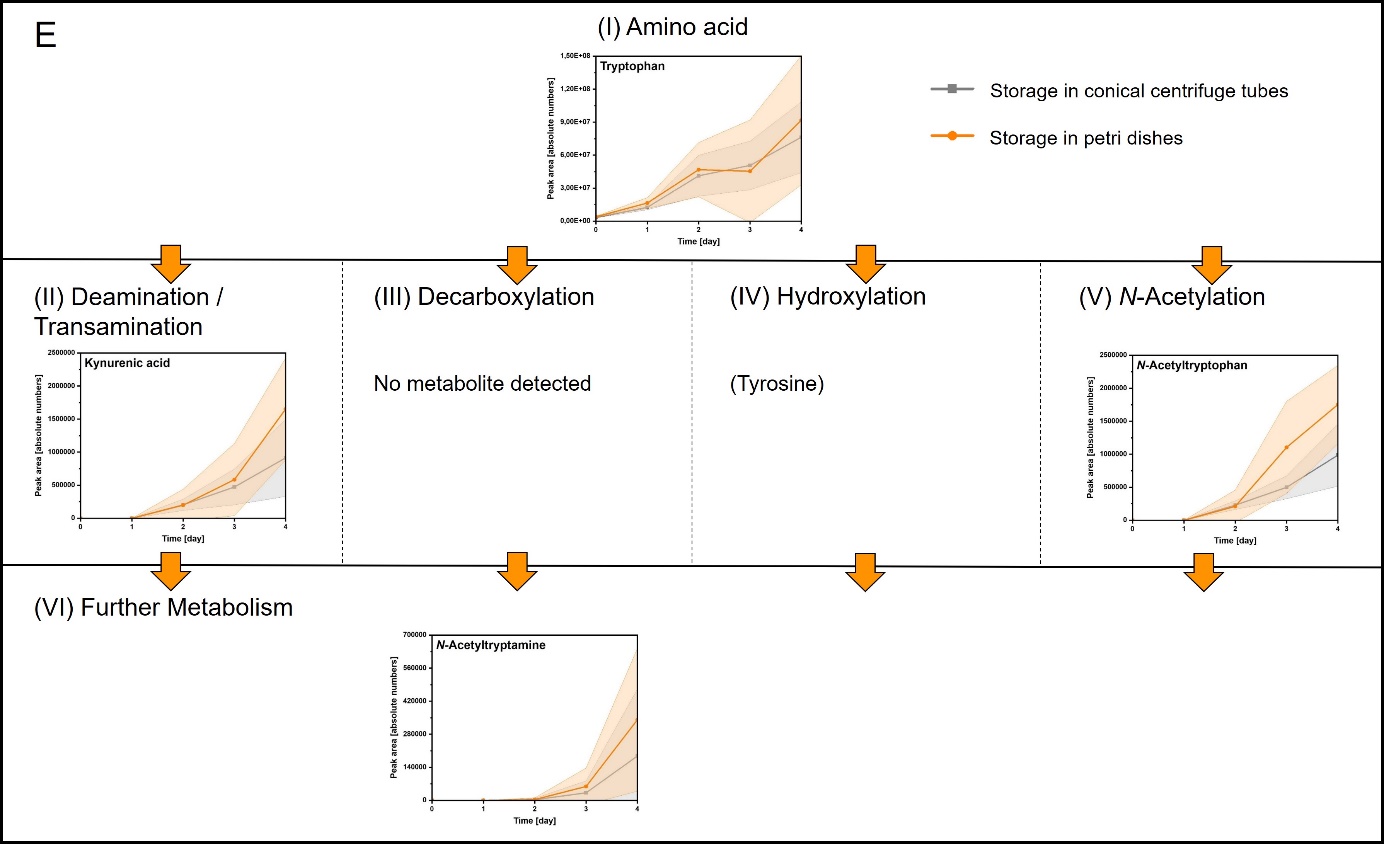
**

***Online Resource 1 – Fig. 3: Detected absolute peak areas for lysine, arginine, tyrosine, phenylalanine, tryptophan and their respective metabolites****. Mean values (lines) and ranges (coloured areas) of absolute peak areas are shown for all five amino acids (I) and their respective deamination/transamination (II), decarboxylation (III), hydroxylation (IV), N-acetylation (V) metabolites and metabolites resulting from further steps of pathways (II) to (V) for both storage conditions in conical centrifuge tubes and petri dishes for the four experiment days. A – lysine and metabolites/degradation products, B – arginine and metabolites/degradation products, C – tyrosine and metabolites/degradation products, D – phenylalanine and metabolites/degradation products, E – tryptophan and metabolites/degradation products*
