# Supplementary material for: Amino acids and their metabolites as potential biochemical markers in postmortem vitreous humour
Source: Int J Legal Med. 2025 Jul 2;139(6):3051–62. doi: 10.1007/s00414-025-03552-9 (PMC12672791; doi:10.1007/s00414-025-03552-9)
Supplement: Supplementary file 2 — (DOCX 54.1 KB) [file 414_2025_3552_MOESM2_ESM.docx]

**Online Resource 2**

International Journal of Legal Medicine

**Amino acids and their Metabolites as potential biochemical Markers in *postmortem* Vitreous Humour**

Laura Franke^1^*, Hannah Ihle^1^*, Kristina Rieger^1^, Viviane Stammer^1^, Senta Niederegger^1^, Dirk K. Wissenbach^1^, Frank T. Peters^1^, Gita Mall^1^

^1^Jena University Hospital, Institute for Forensic Medicine, Friedrich Schiller University Jena, Germany

Author to whom correspondence should be addressed. Email: dirk.wissenbach@med.uni-jena.de

***Online Resource 2 – Table 1: Absolute peaks areas for all 21 amino acids and amino acid metabolites/degradation products.*** *Given are the respective biochemical markers, the experiment day, the sample size N, mean values, standard deviations of peak areas and p values for comparison of both storage conditions in conical centrifuge tubes and petri dishes.*

| **Biochemical Marker** | **Day** | **Storage on conical centrifuge tubes** | | | **Storage in petri dishes** | | | **p value** |
| --- | --- | --- | --- | --- | --- | --- | --- | --- |
|  |  | **N** | **Area Mean** | **Standard Deviation** | **N** | **Area Mean** | **Standard Deviation** |  |
| **Lysine** | d0 | 6 | 3183209 | 509964 | 6 | 3554825 | 424148 | 0.2000 |
|  | d1 | 6 | 5329476 | 733436 | 6 | 5865867 | 438679 | 0.1552 |
|  | d2 | 4 | 29157195 | 14390577 | 4 | 10995106 | 3838533 | 0.0505 |
|  | d3 | 6 | 38330668 | 13619695 | 5 | 39869664 | 14720899 | 0.8611 |
|  | d4 | 6 | 40568665 | 14453646 | 4 | 38614678 | 16160588 | 0.8463 |
| **Histidine** | d0 | 6 | 6350889 | 1374064 | 6 | 8748775 | 2321782 | 0.0545 |
|  | d1 | 6 | 28450633 | 3316050 | 6 | 38693096 | 8576342 | 0.0212 |
|  | d2 | 4 | 63653950 | 25182127 | 4 | 53677358 | 15513041 | 0.5250 |
|  | d3 | 6 | 60239603 | 25802739 | 5 | 78155913 | 24962426 | 0.2746 |
|  | d4 | 6 | 40458510 | 11714379 | 4 | 59233175 | 18763386 | 0.0842 |
| **Arginine** | d0 | 6 | 45276714 | 3895096 | 6 | 47177907 | 4918435 | 0.4750 |
|  | d1 | 6 | 49795132 | 4380499 | 6 | 55979563 | 7526109 | 0.1126 |
|  | d2 | 4 | 67858542 | 15215582 | 4 | 44230167 | 9891425 | 0.0404 |
|  | d3 | 6 | 17207303 | 24335811 | 5 | 103901396 | 48562185 | 0.0039 |
|  | d4 | 6 | 83914975 | 68389444 | 4 | 47697460 | 45466623 | 0.3832 |
| ***N*^2^-Acetyllysine** | d0 | 6 | 39923 | 34623 | d0 | 46396 | 25692 | 0.7207 |
|  | d1 | 6 | 69380 | 46464 | d1 | 58039 | 25700 | 0.6122 |
|  | d2 | 4 | 6924782 | 3988090 | d2 | 2328873 | 2123601 | 0.0881 |
|  | d3 | 6 | 9200981 | 2339946 | d3 | 10070775 | 4255749 | 0.6764 |
|  | d4 | 6 | 18211554 | 10416701 | d4 | 32947646 | 11475453 | 0.0680 |
| ***N*^2^-Acetylarginine** | d0 | 6 | 12318 | 6317 | d0 | 15454 | 4052 | 0.3302 |
|  | d1 | 6 | 104819 | 53167 | d1 | 158524 | 165338 | 0.4663 |
|  | d2 | 4 | 11386198 | 6292093 | d2 | 6508541 | 3029091 | 0.2119 |
|  | d3 | 6 | 11407359 | 1844298 | d3 | 12219621 | 5885975 | 0.7543 |
|  | d4 | 6 | 11061374 | 4087370 | d4 | 22086559 | 7825622 | 0.0183 |
| ***N*^6^-Acetyllysine** | d0 | 6 | 63117 | 9143 | 6 | 49811 | 25449 | 0.2558 |
|  | d1 | 6 | 114856 | 69985 | 6 | 134719 | 62005 | 0.6141 |
|  | d2 | 4 | 2700865 | 3721703 | 4 | 676569 | 919807 | 0.3316 |
|  | d3 | 6 | 5503416 | 4006564 | 5 | 870316 | 314641 | 0.0309 |
|  | d4 | 6 | 2898144 | 1910644 | 4 | 1323826 | 1163306 | 0.1823 |
| ***N*-Acetylcadaverine** | d0 | 6 | 2544 | 1496 | 6 | 742 | 455 | 0.0181 |
|  | d1 | 6 | 52725 | 103158 | 6 | 130633 | 221357 | 0.4527 |
|  | d2 | 4 | 5789717 | 3882089 | 4 | 4506395 | 3813475 | 0.6538 |
|  | d3 | 6 | 19999493 | 27221364 | 5 | 15111853 | 13540753 | 0.7246 |
|  | d4 | 6 | 20661927 | 15552266 | 4 | 52711140 | 47338536 | 0.1535 |
| **Tyrosine** | d0 | 6 | 545966 | 126991 | 6 | 589746 | 103199 | 0.5270 |
|  | d1 | 6 | 1010756 | 151140 | 6 | 1269275 | 263836 | 0.0639 |
|  | d2 | 4 | 4516004 | 2455868 | 4 | 3862554 | 2074496 | 0.6985 |
|  | d3 | 6 | 7880406 | 2830210 | 5 | 9794955 | 3919712 | 0.3710 |
|  | d4 | 6 | 7635611 | 2252178 | 4 | 11791175 | 4327193 | 0.0785 |
| ***N*-Acetylagmatine** | d0 | 6 | not detected | not detected | 6 | 60 | 148 | 0.3441 |
|  | d1 | 6 | 113 | 276 | 6 | 1327 | 3082 | 0.3592 |
|  | d2 | 4 | 688753 | 818822 | 4 | 728444 | 1252486 | 0.9594 |
|  | d3 | 6 | 2393108 | 3574513 | 5 | 896983 | 946492 | 0.3904 |
|  | d4 | 6 | 1851481 | 1542106 | 4 | 1566078 | 1599681 | 0.7846 |
| **Tyramine** | d0 | 6 | not detected | not detected | 6 | not detected | not detected | - |
|  | d1 | 6 | not detected | not detected | 6 | not detected | not detected | - |
|  | d2 | 4 | 13127310 | 9319276 | 4 | 5019020 | 7078394 | 0.2151 |
|  | d3 | 6 | 64817864 | 67141684 | 5 | 13421691 | 12483094 | 0.1286 |
|  | d4 | 6 | 69069615 | 79195218 | 4 | 27465941 | 31929457 | 0.3546 |
| **Phenylalanine** | d0 | 6 | 16311906 | 2111648 | 6 | 20509269 | 2106585 | 0.0063 |
|  | d1 | 6 | 37371945 | 5089033 | 6 | 47728401 | 3961426 | 0.0028 |
|  | d2 | 4 | 268721302 | 146140231 | 4 | 173265234 | 101099316 | 0.3240 |
|  | d3 | 6 | 497759724 | 231725731 | 5 | 532803858 | 222241812 | 0.8050 |
|  | d4 | 6 | 749211324 | 291604050 | 4 | 743343871 | 312195130 | 0.9765 |
| ***N*^2.6^-Diacetyllysine** | d0 | 6 | not detected | not detected | 6 | not detected | not detected | - |
|  | d1 | 6 | 69 | 168 | 6 | 203 | 338 | 0.4049 |
|  | d2 | 4 | 156806 | 44601 | 4 | 99553 | 144622 | 0.4779 |
|  | d3 | 6 | 347836 | 165471 | 5 | 1156946 | 1064578 | 0.0966 |
|  | d4 | 6 | 1959859 | 837090 | 4 | 6010019 | 6162731 | 0.1401 |
| **2-Phenylethylamine** | d0 | 6 | 412 | 157 | 6 | 923 | 599 | 0.0708 |
|  | d1 | 6 | 405 | 137 | 6 | 433 | 230 | 0.8030 |
|  | d2 | 4 | 358623 | 710677 | 4 | 86748 | 163620 | 0.4841 |
|  | d3 | 6 | 1122964 | 2198424 | 5 | 99468 | 215062 | 0.3310 |
|  | d4 | 6 | 1127023 | 2376421 | 4 | 1129377 | 1811396 | 0.9987 |
| **Tryptophan** | d0 | 6 | 3360089 | 365894 | 6 | 4028693 | 458692 | 0.0191 |
|  | d1 | 6 | 12569659 | 2169896 | 6 | 16528109 | 4922432 | 0.1016 |
|  | d2 | 4 | 41262692 | 18620022 | 4 | 46796369 | 24692616 | 0.7327 |
|  | d3 | 6 | 50647044 | 22119727 | 5 | 45353700 | 46570673 | 0.8092 |
|  | d4 | 6 | 76276883 | 32117935 | 4 | 91747766 | 58733473 | 0.6010 |
| **Kynurenic Acid** | d0 | 6 | not detected | not detected | 6 | not detected | not detected | - |
|  | d1 | 6 | not detected | not detected | 6 | not detected | not detected | - |
|  | d2 | 4 | 204072 | 84919 | 4 | 198534 | 241167 | 0.9669 |
|  | d3 | 6 | 473368 | 269862 | 5 | 584744 | 545650 | 0.6686 |
|  | d4 | 6 | 913998 | 583899 | 4 | 1652574 | 768874 | 0.1209 |
| ***N*-Acetyltyrosine** | d0 | 6 | not detected | not detected | 6 | not detected | not detected | - |
|  | d1 | 6 | not detected | not detected | 6 | not detected | not detected | - |
|  | d2 | 4 | 6456 | 7163 | 4 | 3616 | 5736 | 0.5587 |
|  | d3 | 6 | 30690 | 25924 | 5 | 181880 | 197125 | 0.0928 |
|  | d4 | 6 | 84004 | 54249 | 4 | 405304 | 273547 | 0.0206 |
| ***N*-Acetyltyramine** | d0 | 6 | not detected | not detected | 6 | not detected | not detected | - |
|  | d1 | 6 | not detected | not detected | 6 | not detected | not detected | - |
|  | d2 | 4 | 395274 | 286703 | 4 | 130589 | 261178 | 0.2212 |
|  | d3 | 6 | 2938992 | 2041660 | 5 | 1193857 | 1120529 | 0.1233 |
|  | d4 | 6 | 14366962 | 9546568 | 4 | 6002470 | 4917541 | 0.1494 |
| ***N*-Acetylphenylalanine** | d0 | 6 | not detected | not detected | 6 | not detected | not detected | - |
|  | d1 | 6 | not detected | not detected | 6 | not detected | not detected | - |
|  | d2 | 4 | 27465 | 32036 | 4 | 1540 | 3080 | 0.1583 |
|  | d3 | 6 | 70033 | 59806 | 5 | 192915 | 210959 | 0.2022 |
|  | d4 | 6 | 165400 | 132044 | 4 | 805379 | 407034 | 0.0063 |
| ***N*-Acetyltryptophan** | d0 | 6 | not detected | not detected | 6 | not detected | not detected | - |
|  | d1 | 6 | not detected | not detected | 6 | not detected | not detected | - |
|  | d2 | 4 | 228854 | 66468 | 4 | 210500 | 246878 | 0.8905 |
|  | d3 | 6 | 499491 | 174163 | 5 | 1104200 | 698411 | 0.0688 |
|  | d4 | 6 | 987422 | 470521 | 4 | 1754016 | 593317 | 0.0518 |
| ***N*-Acetylphenylethylamine** | d0 | 6 | not detected | not detected | 6 | not detected | not detected | - |
|  | d1 | 6 | not detected | not detected | 6 | not detected | not detected | - |
|  | d2 | 4 | 273220 | 284772 | 4 | 127191 | 230113 | 0.4554 |
|  | d3 | 6 | 1067721 | 827395 | 5 | 1203342 | 960676 | 0.8068 |
|  | d4 | 6 | 10923443 | 12925341 | 4 | 10288788 | 12111183 | 0.9398 |
| ***N*-Acetyltryptamine** | d0 | 6 | not detected | not detected | 6 | not detected | not detected | - |
|  | d1 | 6 | not detected | not detected | 6 | not detected | not detected | - |
|  | d2 | 4 | 3045 | 6090 | 4 | 3647 | 7294 | 0.9033 |
|  | d3 | 6 | 32144 | 50198 | 5 | 59004 | 78164 | 0.5067 |
|  | d4 | 6 | 188047 | 283796 | 4 | 341557 | 302285 | 0.4373 |

***Online Resource 2 – Table 2: p values of each analyte under conical centrifuge tube storage to access significant peak area changes between days.*** *Unpaired. two-tailed t-tests were applied with a significance level of α=0.05.*

| **Analyte** | **d0/d1** | **d0/d2** | **d0/d3** | **d0/d4** | **d1/d2** | **d1/d3** | **d1/d4** | **d2/d3** | **d2/d4** | **d3/d4** |
| --- | --- | --- | --- | --- | --- | --- | --- | --- | --- | --- |
| **2-Phenylethylamine** | 0.9330 | 0.2380 | 0.2395 | 0.2725 | 0.2380 | 0.2395 | 0.2725 | 0.5272 | 0.5542 | 0.9976 |
| ***N*-Acetylagmatine** | 0.3409 | 0.0660 | 0.1321 | 0.0148 | 0.0660 | 0.1321 | 0.0148 | 0.3845 | 0.2090 | 0.7403 |
| ***N*-Acetylcadaverine** | 0.2610 | 0.0055 | 0.1022 | 0.0087 | 0.0057 | 0.1029 | 0.0088 | 0.3390 | 0.1031 | 0.9597 |
| ***N*-Acetylphenethylamin** | - | 0.0414 | 0.0101 | 0.0653 | 0.0414 | 0.0101 | 0.0653 | 0.1065 | 0.1451 | 0.0919 |
| ***N*-Acetyltryptamine** | - | 0.2415 | 0.1478 | 0.1356 | 0.2415 | 0.1478 | 0.1356 | 0.2908 | 0.2373 | 0.2146 |
| ***N*-Acetyltryptophan** | - | 0.0000 | 0.0000 | 0.0004 | 0.0000 | 0.0000 | 0.0004 | 0.0193 | 0.0138 | 0.0385 |
| **Kynurenic acid** | - | 0.0003 | 0.0016 | 0.0033 | 0.0003 | 0.0016 | 0.0033 | 0.0940 | 0.0454 | 0.1243 |
| ***N*-Acetyltyramine** | - | 0.0082 | 0.0055 | 0.0042 | 0.0082 | 0.0055 | 0.0042 | 0.0414 | 0.0209 | 0.0167 |
| **Phenylalanin** | 0.0000 | 0.0024 | 0.0005 | 0.0001 | 0.0039 | 0.0007 | 0.0001 | 0.1200 | 0.0168 | 0.1292 |
| **Tryptophan** | 0.0000 | 0.0009 | 0.0004 | 0.0002 | 0.0048 | 0.0018 | 0.0007 | 0.5059 | 0.0872 | 0.1385 |
| **Tyramine** | - | 0.0074 | 0.0396 | 0.0584 | 0.0074 | 0.0396 | 0.0584 | 0.1720 | 0.2054 | 0.9221 |
| **Tyrosine** | 0.0002 | 0.0035 | 0.0001 | 0.0000 | 0.0070 | 0.0001 | 0.0000 | 0.0893 | 0.0718 | 0.8716 |
| **Arginine** | 0.0883 | 0.0073 | 0.0191 | 0.1972 | 0.0227 | 0.0090 | 0.2506 | 0.0063 | 0.6623 | 0.0481 |
| **Lysine** | 0.0002 | 0.0018 | 0.0001 | 0.0001 | 0.0031 | 0.0001 | 0.0001 | 0.3370 | 0.2554 | 0.7881 |
| ***N*^2^-Acetyllysine** | 0.2414 | 0.0024 | 0.0000 | 0.0016 | 0.0025 | 0.0000 | 0.0016 | 0.2830 | 0.0762 | 0.0656 |
| ***N*^6^-Acetyllysine** | 0.1028 | 0.1107 | 0.0077 | 0.0046 | 0.1169 | 0.0081 | 0.0051 | 0.2982 | 0.9138 | 0.1811 |
| ***N*^2^-Acetylarginine** | 0.0017 | 0.0018 | <0,000001 | 0.0001 | 0.0019 | <0,000001 | 0.0001 | 0.9938 | 0.9228 | 0.8539 |
| ***N*^2,6-^Acetyllysine** | 0.3381 | 0.0000 | 0.0004 | 0.0002 | 0.0000 | 0.0004 | 0.0002 | 0.0577 | 0.0029 | 0.0009 |
| ***N*-Acetyltyrosine** | - | 0.0521 | 0.0158 | 0.0035 | 0.0521 | 0.0158 | 0.0035 | 0.1110 | 0.0237 | 0.0550 |
| ***N*-Acetylphenylalanine** | - | 0.0619 | 0.0167 | 0.0119 | 0.0619 | 0.0167 | 0.0119 | 0.2337 | 0.0791 | 0.1381 |

***Online Resource 2 – Table 3: p values of each analyte under petri dish storage to access significant peak area changes between days.*** *Unpaired. two-tailed t-tests were applied with a significance level of α=0.05.*

| **Analyte** | **d0/d1** | **d0/d2** | **d0/d3** | **d0/d4** | **d1/d2** | **d1/d3** | **d1/d4** | **d2/d3** | **d2/d4** | **d3/d4** |
| --- | --- | --- | --- | --- | --- | --- | --- | --- | --- | --- |
| **2-Phenylethylamine** | 0.0907 | 0.2211 | 0.2857 | 0.1537 | 0.2188 | 0.2834 | 0.1535 | 0.9251 | 0.2952 | 0.2404 |
| ***N*-Acetylagmatine** | 0.3382 | 0.1794 | 0.0435 | 0.0383 | 0.1801 | 0.0437 | 0.0384 | 0.8240 | 0.4412 | 0.4574 |
| ***N*-Acetylcadaverine** | 0.1812 | 0.0174 | 0.0220 | 0.0226 | 0.0201 | 0.0228 | 0.0228 | 0.1772 | 0.0887 | 0.1296 |
| ***N*-Acetylphenethylamin** | - | 0.1996 | 0.0127 | 0.0639 | 0.1996 | 0.0127 | 0.0639 | 0.0673 | 0.1444 | 0.1327 |
| ***N*-Acetyltryptamine** | - | 0.2415 | 0.0943 | 0.0212 | 0.2415 | 0.0943 | 0.0212 | 0.2065 | 0.0668 | 0.0808 |
| ***N*-Acetyltryptophan** | - | 0.0631 | 0.0035 | 0.0001 | 0.0631 | 0.0035 | 0.0001 | 0.0466 | 0.0030 | 0.1830 |
| **Kynurenic acid** | - | 0.0708 | 0.0263 | 0.0006 | 0.0708 | 0.0263 | 0.0006 | 0.2336 | 0.0112 | 0.0444 |
| ***N*-Acetyltyramine** | - | 0.2415 | 0.0269 | 0.0149 | 0.2415 | 0.0269 | 0.0149 | 0.1093 | 0.0544 | 0.0683 |
| **Phenylalanin** | <0.000001 | 0.0051 | 0.0003 | 0.0004 | 0.0139 | 0.0004 | 0.0005 | 0.0209 | 0.0132 | 0.2742 |
| **Tryptophan** | 0.0001 | 0.0023 | 0.0555 | 0.0054 | 0.0170 | 0.1622 | 0.0122 | 0.9573 | 0.2079 | 0.2263 |
| **Tyramine** | - | 0.1106 | 0.0259 | 0.0612 | 0.1106 | 0.0259 | 0.0612 | 0.2723 | 0.2189 | 0.3917 |
| **Tyrosine** | 0.0002 | 0.0040 | 0.0003 | 0.0002 | 0.0142 | 0.0004 | 0.0003 | 0.0301 | 0.0163 | 0.4914 |
| **Arginine** | 0.0374 | 0.5435 | 0.0183 | 0.9779 | 0.0644 | 0.0393 | 0.6642 | 0.0484 | 0.8864 | 0.1195 |
| **Lysine** | 0.0000 | 0.0013 | 0.0002 | 0.0006 | 0.0102 | 0.0003 | 0.0009 | 0.0070 | 0.0159 | 0.9064 |
| ***N*^2^-Acetyllysine** | 0.4508 | 0.0263 | 0.0002 | 0.0001 | 0.0269 | 0.0003 | 0.0001 | 0.0132 | 0.0019 | 0.0042 |
| ***N*^6^-Acetyllysine** | 0.0112 | 0.1232 | 0.0001 | 0.0243 | 0.1759 | 0.0003 | 0.0326 | 0.6690 | 0.4163 | 0.4248 |
| ***N*^2^-Acetylarginine** | 0.0601 | 0.0006 | 0.0006 | 0.0001 | 0.0007 | 0.0007 | 0.0001 | 0.1240 | 0.0099 | 0.0668 |
| ***N*^2,6-^Acetyllysine** | 0.1720 | 0.1198 | 0.0247 | 0.0389 | 0.1204 | 0.0247 | 0.0389 | 0.0928 | 0.1036 | 0.1221 |
| ***N*-Acetyltyrosine** | - | 0.1494 | 0.0481 | 0.0056 | 0.1494 | 0.0481 | 0.0056 | 0.1178 | 0.0261 | 0.1959 |
| ***N*-Acetylphenylalanine** | - | 0.2415 | 0.0497 | 0.0010 | 0.2415 | 0.0497 | 0.0010 | 0.1168 | 0.0075 | 0.0217 |
